# Diabetes prevalence and risk factors, underestimated without oral glucose tolerance test, in rural Gombe-Matadi Adults, Democratic Republic of Congo, 2019

**DOI:** 10.1038/s41598-022-18658-y

**Published:** 2022-09-12

**Authors:** Muel Telo Marie-Claire Muyer, Steve Botomba, Nickson Poka, Dieudonné Mpunga, Deogratias Katsuva Sibongwere, José Luis Peñalvo, Diana Sagastume, Mala Ali Mapatano

**Affiliations:** 1 Department of Nutrition, School of Public Health, Epidemiology Center of Diabetes, Kinshasa, Democratic Republic of Congo; 2 Education Center of Diabetes and Health, Epidemiology Center of Diabetes, Kinshasa, Democratic Republic of Congo; 3 Department of Community Health, School of Public Health, Kinshasa, Democratic Republic of Congo; 4 Department of Nutrition, School of Public Health, Kinshasa, Democratic Republic of Congo; 5 Non-Communicable Diseases Unit, Department of Public Health, Institute of Tropical Medicine of Antwerp, Sint-Rochusstraat 43, 2000 Antwerp, Belgium; 6 Education Department, Institute of Tropical Medicine of Antwerp, Sint-Rochusstraat 43, 2000 Antwerp, Belgium

**Keywords:** Diseases, Endocrinology, Risk factors

## Abstract

An increase in the diabetes prevalence is reported worldwide. We aimed to determine the diabetes prevalence and its risk factors among adults in a rural area of the Democratic Republic of Congo. A cross-sectional study was conducted in 1531 inhabitants, selected by five stages, in the Health Zone of Gombe-Matadi. Diabetes was defined according to the American Diabetes Association and the International Diabetes Federation. Fasting glycemia and/or an oral glucose tolerance test were collected. We measured body mass index, waist circumference and blood pressure. Mann Whitney's and chi-square tests compared respondents with non-respondents. Multivariable logistic regression measured associations between diabetes and its risk factors. Crude and standardized prevalence of diabetes were 6.7% and 5.3%, respectively. Undiagnosed diabetes accounted for 58.8%. The oral glucose tolerance test alone diagnosed 2.6% of cases. Diabetes was more frequent in males, unemployed, obese and hypertensive (*p* < 0.05). Risk factors for diabetes were being male, aged ≥ 40 years, general and abdominal obesity associated with elderly, family history of diabetes, and hypertension. Diabetes in rural areas of the Democratic Republic of Congo appears to be underdiagnosed. The oral glucose tolerance test provides an opportunity to screen individuals for diabetes in this setting.

## Introduction

Globally, it has been estimated that between 1995 and 2025, diabetes mellitus will have recorded 170% of increase^1^. According to the International Diabetes Federation (IDF), in 2019, three-fourths of patients with diabetes lived in low-income countries^2^. Currently, diabetes is experiencing an accelerated increase in Africa as in other developing areas^3^. This is attributed to various factors, such as fast economic development and urbanization^4^, leading to rapid changes of the population towards unhealthy lifestyles, such as lack of physical activity and sub-optimal diets, leading to excess weight and the onset of cardiometabolic risk factors. Gender also plays a role in this increase. Some studies shows a higher risk for type 2 diabetes in adult men^5^ and a higher type 2 diabetes prevalence, mainly in older men. This was associated with differences in visceral fat accumulation compared to women^6^. The same situation was found in South Africa wherethis adiposity was linked to decreased insulin sensitivity and beta-cell function^7^.

Diabetes is currently counted among the 10 leading causes of death worldwide and along with the other major non-communicable diseases namely cardiovascular diseases, cancer, and chronic respiratory diseases, accounts for more than 80% of all premature deaths^8^. Moreover, it is estimated that 37.8 to 59.7% of patients with diabetes worldwide go undiagnosed, Sub-Saharan Africa having the greatest burden with 59.7% of undiagnosed cases^2^. In line with this, it has been reported that more than 75% of countries in Africa do not have epidemiological data on the prevalence of diabetes in adults^3^, and most available data often concern urbanized settings. On the other hand, more undiagnosed cases have been observed in rural areas^9^, likely because of the lack of financial resources to carry out these studies. The delay of diabetes diagnosis leads to severe chronic complications, such as retinopathy, nephropathy, peripheral neuropathy^10^, peripheral vascular disease, ischemic heart disease, and stroke^11^.

In 2019, diabetes prevalence was estimated at 9.3% worldwide^2^. In African rural areas, it was estimated at 2.4%^2^, ranging from 0% in Togo in 1987^12^ to 16% in Uganda in 2016^9^. The factors underlying this increase were particularly abdominal obesity^13^, smoking and hypertension^9^.

In the Democratic Republic of Congo (DRC), no nationwide study has been conducted on diabetes prevalence. The prevalence in urban areas of DRC varied from 11.7 to 15.5% in 2006^14,15^, while in rural and semi-rural areas a much lower prevalence has been reported ranging between 1.7% in the East and 4.8% in the West^16–18^ from 2007 to 2015. The risk factors for diabetes identified in the East were aging, abdominal obesity, and hypertension, whereas in the West, aging, abdominal obesity, being male, and history of diabetes in the family were more associated with prevalent diabetes.

Since 2015, no study has explored the prevalence of diabetes in rural areas. Of the 516 Health zones (HZs) of DRC, few health facilities have integrated activities regarding diabetes, being one of them the Gombe Matadi HZ, located in the Kongo Central province and 188 km away from Kinshasa, the capital city of DRC. This study aims to determine the prevalence of diabetes and its risk factors among adults in Gombe-Matadi Rural HZ.

## Methods

### Study design

This was a population-based cross-sectional survey targeting households.

### Settings

**Figure 1:**
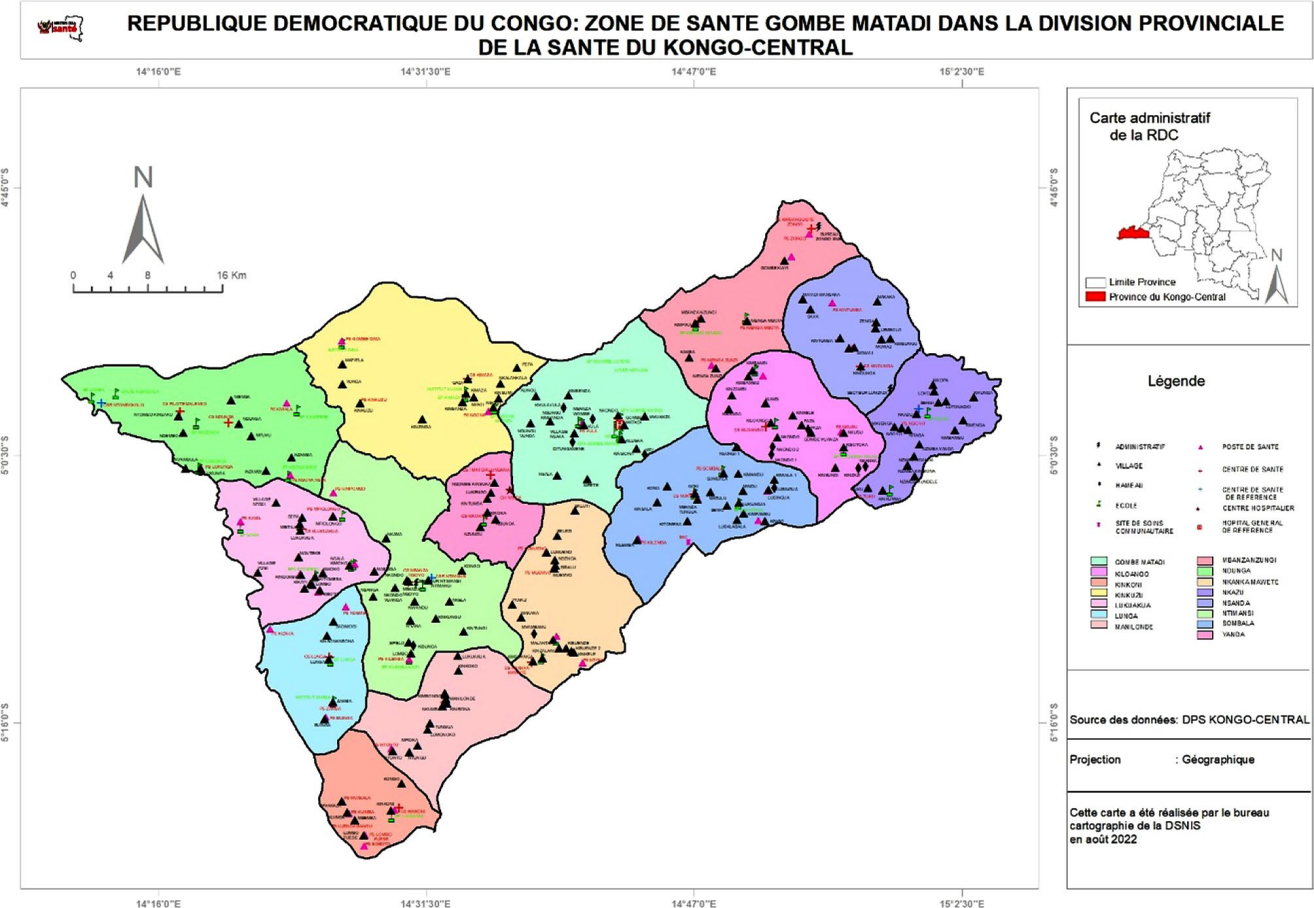
The map of Gombe Matadi Health Zone (*Source*: The Health Zone of Gombe Matadi, Software QGIS 3.4 2018, https://www.qgis.org).

The study was conducted in May 2019 in the Gombe-Matadi Rural HZ which has 15 health areas (HA) (Fig. 1). The study was carried in Gombe-Matadi (26 villages), Yanda (26 villages), and Ntimansi (25 villages) HA. Ntimansi is a strictly rural environment, Gombe Matadi a commercial place serving as transit for travelers from Kinshasa, and Yanda (strictly rural) a religious environment due to the presence of the city of Nkamba (the international headquarters of the Kimbanguiste church) and also influenced by international visitors.

### Study population

The eligibility criteria included individuals with 19 years of age or above^19^ (the risk of diabetes in the youngest age is minimal), both sexes, and have signed the consent form. Pregnant women (defined by amenorrhea of more than 2 months for women younger than 46 years, outside exclusive breastfeeding), individuals with severe psychiatric conditions, non-Congolese and non-resident individuals were excluded.

### Sampling

The methodology followed is the one proposed by the World Health Organization (WHO)^19^ and the American Diabetes Association (ADA)^20^ which allows for standardization of methods and comparison with other countries.

A sample of 1600 individuals was obtained by the following formula: **n = (Z**^**2**^**pq/d**^**2**^)  * g * 1/1 − f. ***Z***_***95%***_ coefficient was equal to 1.96. The prevalence of diabetes, ***p*** = 0.05 (***q*** = 1 − *p*), corresponded to that found in Kisantu, in DRC^18^. The desired degree of precision ***d*** was equivalent to 0.02, and ***g*** represented the correction coefficient for the cluster effect estimated at 2^21^. We used a proportional sample (Table 1). The fraction of non-responses ***f*** was estimated at 10% .

**Table 1:** Proportional sampling according to health area. *Source*: Central office of Gombe-Matadi health zone, 2018^18^.

Health area	Total population	Proportion	Proportional sample of population
Gombe-Matadi	6450	29.8	477
Yanda	6493	30.0	480
Ntimansi	8716	40.2	643
Total	21,659	100	1600

To select the survey participants, we proceeded in five stages^22,23^. In the first stage, three HA selected by simple random sampling were considered as clusters. In the second stage, villages within clusters were selected according to demographic weight (> 200 inhabitants) and distance (< 4 km from the study site). In the third stage, a systematic drawing of the inhabited plots in each selected village after having listed and numbered all the inhabited plots was performed. The sampling step for each HA village was the number of inhabited plots (N) divided by the proportional sample of the village (n). All the plots identified were chosen. In the fourth degree, after having listed all households, a simple random selection of households was carried out in each plot. In the fifth degree, an eligible subject was drawn by simple random selection from each household.

### Operational definitions

Diabetes was defined according to the ADA 2018^20^ and the WHO/International Diabetes Federation 2006^24^, on the basis of, for a new case at least two altered glycaemia (fasting plasma glucose (FPG) ≥ 126 mg/dLL) and/or oral glucose tolerance test (OGTT) ≥ 200 mg/dL. For a known case, a diabetic notebook or an anti-diabetic treatment was considered. The impaired fasting glycaemia (IFG) was defined by an FPG between 100–125 mg/dL on day one or two and an OGTT < 200 mg/dL. An impaired glucose tolerance (IGT) was defined by an FPG < 126 mg/dL on day one and an OGTT between 140–199 mg/dL.

Risk factors were defined as this. General obesity was defined as a body mass index (BMI) (weight (Kg)/ height (m^2^)) ≥ 30 kg/m^2^^25^ and abdominal obesity by a waist circumference measurement ≥ 94 cm and ≥ 80 cm, respectively for men and women^26^. Hypertension was confirmed on the basis of two consecutive measurements of systolic blood pressure (SBP) value ≥ 140 mmHg and diastolic blood pressure (DBP) ≥ 90 mmHg^21,27^, or self-reported. Age was categorized into < 40 years old and ≥ 40 years old, education in low school level (none and primary) and high school level (the other levels), and the usual mode of mobility in a group with physical exercises (foot and bike) and non-physical exercises (motorbike, car). General obesity as well as abdominal obesity in obese and non-obese and the profession in unemployed (student, unemployed, housewife, without profession, disabled, retired) and employees (employed in the state, employed in the private sector, self-employed, farmer, poultry farmer, volunteer, domestic) were also considered. Other binary variables were categorized in yes/no, family history of diabetes, hypertension, macrosomia, alcohol consumption, and smoking.

#### Data collection

Authorizations for the investigation were obtained from the political and administrative authorities and a census of inhabited compounds was performed. The investigation lasted 39 days. Ten field workers recruited among nurses followed a 3-day training course on diabetes, hypertension, the questionnaire, and the census of inhabited compounds. After evaluation, six field workers were selected. A pre-test was organized for all team in an HA of Gombe-Matadi not selected for the survey, on 25 individuals.

The census of inhabited compounds identified during 3 days all the plots by proceeding area by area. In each area the whole team worked together before going to another area. The coordinator and three supervisors came from the Kinshasa School of Public Health.

Fixed study sites were defined to avoid mobilizing too much human and material resources and to improve data quality^20,23^. We chose 3 sites (health center, school or church) by HA. Registration of participants, and first FPG were performed at home after obtaining their consent. An appointment card was provided to the participant for the OGTT, to answer the questionnaire, to take measurements and do FPG in case of not fasting. In the absence of the chosen individual, the field worker returned the same or the next day. No refusal was noted. In the case the elected individual does not meet the criteria, another participant will be drawn randomly. One plot without eligible individuals was skipped and the next was taken. The coding of names was carried out in the evening by the team, by assigning each name a specific code and copying it into their questionnaire.

At the same time, the population was sensitized through the churches, district leaders and community health workers. The team was divided into 2 groups of 3 field workers and 1 supervisor to work in the first 2 sites before ending up all together in the last site. The investigation did not disturb usual work within the site. A snack was provided to avoid the impatience of the hungry participants.

On the first day an FPG was carried out at home, the following day a second FPG was assessed as well as the OGTT, and the interview and measurements were carried out on the study site. Those who did not have their FPG at home (not fasting) had it on the first day at the site and the OGTT the next day. The community health workers recovered the absents from their homes.

After the calibration of devices every morning, the glycemia was assessed using a Godefree glucometer (witch references on plasma). The OGTT was performed every day except for 25 people, two individuals with a glycemia ≥ 200 mg/dL on day one, two individuals with a glycemia ≥ 200 mg/dL on day two, and 21 who were previously diagnosed cases of diabetes. A capillary glycemia was taken and the subject orally ingested 75 g of anhydrous glucose in 250 mL of water for 5 min. After 2 h another capillary glycemia was taken. In total, each participant underwent 3 glycemia tests.

#### Statistical analyses

Data quality checks were performed daily by the supervisors’ team. Data management and analyses were performed with IBM’s SPSS 23.0 statistical software. Descriptive analyses provided the measures of frequency, central tendency and dispersion. The chi-square has compared the proportions. The differences between the non-respondents were compared to the respondents (age, sex, first-day glycemia). Age-standardized prevalence was calculated using the standard population of Doll et al.^28^. The inferential statistics were based on 95% CI and Mann Whitney's test which compared the medians of glycaemia between respondents and non-respondents. Multivariable logistic regression was used to explore the association between diabetes and its risk factors (age < 40 and ≥ 40 years, education, usual physical activity, alcohol consumption, smoking, general obesity, family history of diabetes, occupation, hypertension, and sex). A test was considered statistically significant when *p* was < 0.05.

### Ethic statement

The study was approved by the National Committee for Ethics of DRC, under the number 104/CNES/BN/PMMF/2018 of 23/01/2019. All participants gave their informed consent and the data were confidentially kept. Patients were treated and referred to health centers for follow up.

We confirm that all methods were carried out in accordance with relevant guidelines and regulations.

## Results

**Figure 2:**
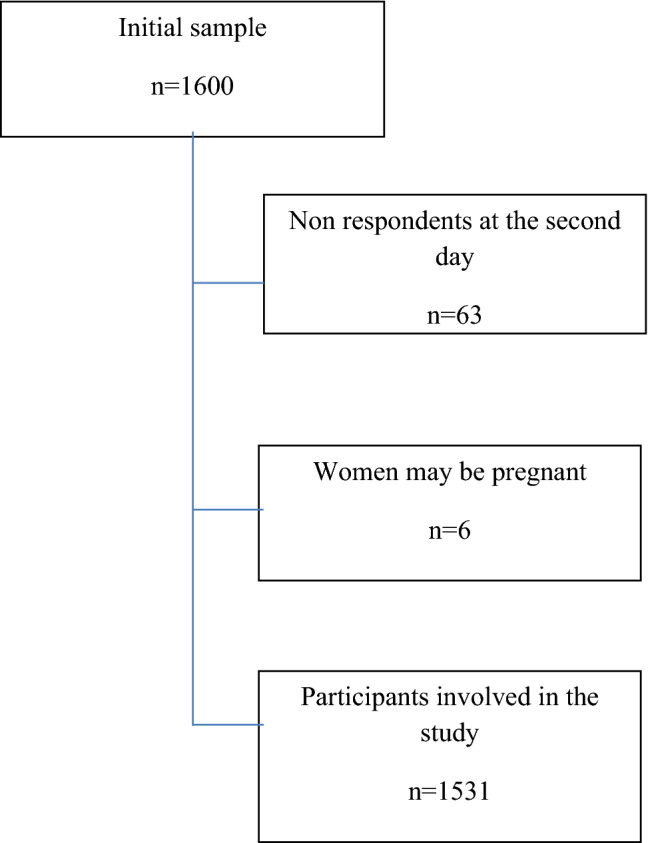
Selection of the study population.

Among the 1600 individuals included in the study, information from 6 (0.4%) women with suspected pregnancy, and 63 (3.9%) participants that did not attend the second day of data collection (non-respondents) were removed. The final dataset contained information from 1531 participants (Fig. 2). Compared to respondents, non-respondents were younger (average age 44.6 ± 12.3 vs. 49.7 ± 14.6 years; *p* = 0.034) while no differences in gender (male 57.1% vs. 47.2%; *p* = 0.955), or the first glycemia (median 96.5 mg/dL IQR 16.8 mg/dL vs. 94.0 mg/dL EQ 15.0 mg/dL; *p* = 0.144) was observed. Among non-respondents, 15 individuals had no glycaemia values and only two had a FPG ≥ 126 mg/dL (137 mg/dL and 316 mg/dL).

### General characteristics of population

Among participants, 39.1% came from Ntimansi, 29.8% from Gombe Matadi and 31.1% from Yanda (Table 1). Around 73.5% of the population were at least 40 years old (minimum 19 years and maximum 96 years), the majority of participants were married (64.5%) and they were slightly more women than men (52.8% vs. 47.2%; *p* < 0.05). Generally, women were less educated than men, illiterate (4.2% vs. 0.9%; *p* < 0.05), more dropped out in primary school (17.8% vs. 9.0%; *p* < 0.05) and few in high school (31.5% vs. 44.1%; *p* < 0.05). The unemployed were 7.8%, while 68.1% were farmers. Among all the participants, 28,4% consumed alcohol, 25% smoke tobacco, 3.7% had general obesity, 32.1% abdominal obesity and hypertension occurred in 20.4% of the participants (Table 2).

**Table 2:** Characteristics of the population of Gombe Matadi.

Characteristics	n	(%)
**Age (years)**		
< 40	417	27.2
≥ 40	1114	72.8
Total	1531	100
**Ethnicity**		
Kongo central	1460	95.4
Others	71	4.6
Total	1531	100
**Health areas**		
Ntimansi	599	39.1
Gombe matadi	456	29.8
Yanda	476	31.1
Total	1531	100
**Gender**		
Male	722	47.2
Female	809	52.8
Total	1531	100
**Marital status**		
Single	151	9.9
Married	987	54.5
Divorced	155	10.1
Widower	203	13.3
Free union	34	2.2
Missing	1	0.1
Total	1531	100
**Profession**		
Employee	1412	92.2
Unemployed	119	7.8
Total	1531	100
**Education**		
Low school level	489	31.9
High school level	1042	68.1
Total	1531	100
**Usual mode of mobility**		
Non-physical exercises	25	1.6
With physical exercises	1506	98.4
Total	1531	100
**Family history of diabetes**		
Yes	242	15.8
No	1289	84.2
Total	1531	100
**General obesity**		
Non obese	1474	96.3
Obese	57	3.7
Total	1531	100
**Abdominal obesity**		
Yes	492	32.1
No	1039	67.9
Total	1531	100
**Alcohol consumption**		
Yes	435	28.5
No	1096	71.6
Total	1531	100
**Tobacco**		
Yes	383	25.0
No	1148	75.0
Total	1531	100
**Hypertension**		
Yes	312	20.4
No	1219	79.6
Total	1531	100

### The prevalence of disglycemia

#### Diabetes mellitus

Crude prevalence of diabetes in the region of Gombe Matadi was 6.7% [95% CI 5.4–7.9]), 3.9% (n = 59 [95% CI 2.9–4.9]) in males and 2.8% [95% CI 2.0–3.7]) in females, *p* = 0.025 (Table 3). Standardized prevalence was 5.3% according to Doll. Two cases of potential diabetes could have been added among the non-respondents (glycaemia day one 137 and 316 mg/dL), but they did not meet our operational definition. Previously diagnosed cases represented 41.2% (n = 42) of the total diabetics and 2.7% (n = 42) of the sample. Undiagnosed diabetes accounted for 58.8% (n = 60) of patients and 3.9% of the sample. Those diagnosed with only two FPG, 0.9%, represented 35% of those diagnosed with OGTT alone (2.6%). The OGTT cases represented 66.7% of new cases, while those who were diabetic at the same time (0.4%) by FPG and OGTT represented 10.3% of new cases. In those who have a FPG < 100 mg% (n = 1040), the prevalence of diabetes by OGTT was 1.3% (n = 13), so half of cases diagnosed with OGTT alone. Reported to all new cases, these represented 12.7%. The médiane of the duration of diabetes in previously diagnosed cases was 18 months, minimum was 2 months and maximum 8 years. Only 51.3% were treated, among them 54.5% used oral anti-diabetics.

**Table 3:** Dysglycemia in the population of Gombe Matadi (n = 1531 participants).

Categories of disglycemia	Total	Male	Female	*p*
	n (%)^a^	n (%)^b^	n (%)^b^	
**Diabetes**				
Total diabetics patients	102 (6.7)	59 (3.9)	43 (2.8)	0.025
Non diabetics	1429 (93.3)	666 (43.3)	766 (50.0)	
Old cases	42 (2.7)	29 (1.9)	13 (0.8)	0.055
New cases	60 (3.9)	30 (2.0)	30 (2.0)	
FPG	14 (0.9)	7 (0.5)	7 (0.5)	0.216
OGTT	40 (2.6)	18 (1.2)	22 (1.4)	
FPG and OGTT combined	6 (0.4)	5 (0.3)	1 (0.1)	
**Intermediate hyperglycemia**				
Total				
Yes	403 (26.3)	187 (12.2)	216 (14.1)	0.723
Non	1128 (73.7)	535 (34.9)	593 (38.7)	
IFG	178 (11.6)	98 (6.4)	80 (5.2)	0.008
IGT	225 (14.7)	89 (5.8)	136 (8.9)	

^a^% according to the total of the sampling.

^b^% according to the column of total per lign.

#### Intermediate hyperglycemia

Among all participants, 11.6% had an IFG and 14.7% had an IGT (*p* = 0.008) suggesting that the total intermediate hyperglycemia amounted to 26.3% and the overall dysglycaemia to 33.0%. Important to point out that the prevalence of IFG was a few higher in male (55.6%) and between the ages of 31–60 years-old (65.2%), while that of IGT was higher in female (60.4%) and between 41 and 70 years-old (63.6%).

#### Bivariate analyzes

In Yanda 53.9% (n = 55) of patients with diabetes were observed. The proportion of patients increased from 3.9% in the 19 to 30 years-old group to 15.7% in the 41 to 50 years-old group. However, 33.3% of the patients were aged between 51 and 60-years old. Males were the majority among patients with diabetes (57.8% vs. 46.4%, *p* < 0.05). The proportion of married was slightly higher among diabetics (69.6% vs. 64.1%, *p* > 0.05) and the proportion of the unemployed among patients with diabetes was more than double than patients without diabetes (17.6% vs. 7.1%, *p* < 0.05). The alcohol and tobacco consumption were almost lower among patients with diabetes when compared to people without diabetes, for alcohol (12.7% vs. 29.5%, *p* < 0.05), and for tobacco (16.7% vs. 25.6% *p* > 0.05). The history of macrosomia in women was almost equivalent in the two groups 86.3% versus 85.2%, *p* > 0.05.

Abdominal obesity was more prevalent, and more pronounced among patients with diabetes compared to without diabetes (55.9% vs. 30.4%, *p* < 0.05). In patients with diabetes and abdominal obesity, 56.1% (n = 32) were males and 43.9 (n = 25) were females, *p* > 0.05. Overall obesity was 9.8% versus 3.3%, *p* < 0.05. The proportion of those with previous hypertension was higher in the group of individuals with diabetes (27.5% vs. 10.4%, *p* < 0.05) than without diabetes. Among diabetic patients, 29.4% (n = 30) were hypertensive at the time of the survey, and among these 33.3% (n = 10) were undiagnosed. Half of the diabetic patients were farmers (50%) compared to 69.5% in the non-diabetic group, *p* < 0.05.

#### Risk factors

A model include general obesity with risk factors variables described in the methods section. In a second model, abdominal obesity was included plus the same variables stated in the previous model. The independent factors for diabetes (Tables 4, 5) were male sex, age ≥ 40 years, and the presence of hypertension. Obesity itself, general or abdominal, was not a risk factor, however, in the elderly, were shown to be a risk factor. Being employed in men decreased the risk of diabetes. Among diabetic patients (n = 102) the unemployed were 17.6% (n = 18) and among the non-diabetics (n = 1429) they were 101 7.1% (n = 101), (*p* < 0.05). Among unemployed diabetics, 3 out 18had general obesity and 15 out 18had abdominal obesity; while the majority, 12 out 18was male.

**Table 4:** Risk factors for diabetes in the population of Gombe Matadi.

Risks factors	OR [IC 95%]	*p*
**Sex**		
Male	4.75 [2.22–10.15]	< 0.001
Female	1	
**Age (years)**		
≥ 40	2.99 [1.51–5.90]	0.002
< 40	1	
**Family history of diabetes**		
Yes	1.81 [1.11–2.92]	0.160
No	1	
**Hypertension**		
Yes	1.59 [1.01–2.52]	0.044
No	1

**Table 5:** Risk factors for diabetes in the population of Gombe Matadi.

Factors	Crude OR	CI	*p*	Adjusted OR	CI	*p*
**Age (years)**						
< 40	1			1		
≥ 40	3.66	1.88–7.11	0.000	2.99	1.51–5.90	0.002
**Sex**						
Female	1			1		
Male	1.58	1.05–2.38	0.026	4.75	2.22–10.15	< 0.001
**Education**						
Low school level	1					
High school level	1.08	0.69–1.67	0.729	–	–	–
**Mode of movement**						
With physical exercice	1					
Without physical exercice	0.8	0.19–3.51	0.787	–	–	–
**Alcohol**						
No	1					
Yes	0.35	0.19–0.63	0.000	–	–	–
**Smoking**						
No	1					
Yes	0.58	0.34–0.99	0.046	–	–	–
**General obesity**						
No	1					
Yes	3.79	1.56–6.53	0.001	–	–	–
**Abdominal obesity**						
No	1					
Yes	2.89	1.92–4.34	0.000	–	–	–
**Family history of diabetes**						
No	1	1.20–3.07	0.006	1	1.11–2.92	0.16
Yes	1.92			1.81		
**HTA**						
No	1			1		
Yes	2.5	1.64–3.81	0.000	1.59	1.01–2.52	0.044

## Discussion

This study aimed to determine the prevalence of diabetes and its risk factors in people aged at least 19 years in a rural area of DRC. The prevalence of diabetes was 6.7%. OGTT has diagnosed the majority of new cases. Risk factors that seem to be associated with diabetes were male sex, year ≥ 40, general and abdominal obesity in elder individuals, family history of diabetes, and hypertension.

The majority of female found here is coherent to the previous data in DRC^29^. May be, males could prefer to deal with their business. The study show also that there were few illiterate people, more among female. Could it be a cultural problem? The unhealthy lifestyle (alcohol) was still strongly noted in this population, which could possibly explain the high prevalence of abdominal obesity in this population. Hypertension was found in 20.4% of participants, being lower than the one found in others African rural areas studies: 21.1% in Mali (2013)^30^, 31% in Cameroon (2013)^31^, 44% in Nigeria (2015)^32^ and 46.9% in Zambia (2017)^33^. The difference between our results and those found through these studies could be explained by the fact that in the majority of these, around more than half of the interviewees were either obese or overweight. Obesity is known to be one of the risk factors for hypertension. However, in our study, these two categories represented less than half of the interviewees. Another reason could be the significant physical activity to which residents of Gombe Matadi are exposed, as a result of the enormous distances they cover in the course of their daily lives.

The few cases of potential diabetes among non-respondents suggests a negligible effect on the prevalence of diabetes, if added, the prevalence will be at 6.8%. The prevalence of diabetes was higher in males than females, as noted by IDF^2^, in contrast with India^34^. The new cases of women had poor exposure to health centers, low educational level and poor health care seeking^34^. In DRC, rural areas have shown so far low prevalence, 1.7% in 2008^17^ and 2.8% between 2012 and 2015^16^ in the East, in Kivu, but higher in the West, 4.8% in 2007 at Kongo Central in Kisantu^18^. Compared to 2007, western rural areas nowadays show an increased prevalence. Note that Kisantu is a semi-rural area where the prevalence was attributable to older age. The diagnosed cases represented 3,4% of cases, because there, diabetes care was already well organized for many years. The higher prevalence in this study can be attributed to new diabetes diagnosed by OGTT. In Kisantu, OGTT was only done in the group of IFG, while in this study it was carried out in all participants. In Sudan in 2013, the undiagnosed diabetes prevalence was more lower, 2.6%^35^ among 1100 individuals. Noor used PFG and casual glycemia. In 2015 in rural Kenya diabetes prevalence was 1.9%. The author used a fasting blood glucose or a self-report of previous diagnosis of diabetes^36^. In rural Uganda, Chiwanga in 2016 noted a prevalence of 16.6%^9^. From a sample size of 200 participants, using only one FPG or self-reported diabetics he found 25 diabetics.

The present study, as Katchunga nine years ago in rural Kivu^17^, reveals a majority of undiagnosed cases of diabetes. This shows a lack of awareness in this population and poor management of diabetes in the community. These ignored patients with diabetes will consult health centers with complications of the disease, increasing the burden of the disease. Also, this means a probably lack of training among healthcare providers to diagnose the diabetes^9^ and also lack of population awareness on how to identify signs.

Only 51.3% patients with diabetes were on treatment (among the 42 known cases, 12 were on oral diabetic traitment, 4 on insulin and 3 on indigene medicine and 3 were on diet alone), The majority of known cases were found in Yanda, in Nkamba village wherethe believers come from everywhere to search for miracle solutions for their illnesses.

The high prevalence of diabetes ranges between 51 and 60 years following the tendency of the Western countries^3^, opposite to Senegal where the higher prevalence is between 18 and 34 years^37^. The large proportion of non-diabetics with a family history of diabetes could predict an increase in cases.

Two-thirds of the new patients were diagnosed using OGTT. However, usually, the diagnosis is made only with the FPG, leaving a gap of two thirds of undiagnosed patients. On the other hand, diabetes diagnosed with OGTT exclusively is associated with a worse prognosis^24^ in terms of mortality and complications, especially diabetic retinopathy. Postprandial hyperglycaemia has been proposed to be a risk factor for cardiovascular disease, stroke, retinopathy, kidney disease and neurological complications^13^. One of the proposed mechanisms of diabetic vascular disease is the oxidative stress which increases after a meal having a high glycemic index^38^.

Previous studies have shown an underestimation of cases^14,17,18^. OGTT was not or partially performed, thereby indicating that the real revalence of diabetes in DRC is higher than our findings. In 2007 our research team^18^, have realised the OGTT only to those whose FPG was higher than 100 mg/dL. In this study, the diabetes found in this group represented 12.7% of all new cases. This implies that, despite the drawbacks of this exam diagnostic test, a solution must be found to include it in screening.

The literature describes a higher prevalence in women^24^, as in our study. Women are likely to have more time to consult while men are at work. In our country and in Gombe Matadi Health Zone specifically, women generally consult health care facilities more than men, even if they go to field or to small market. The National Health Information System reveals the same problem for most of the country's health zones and Gombe Matadi too^39^. Unlike other data showing an increased prevalence with age, our study presents an increasing prevalence with age and then a regression in older ages. The low life expectancy at older ages could explain this. The high prevalence of IGT suggests an increasing rate of diabetes, knowing that after a few years apart of this IGT can shift to diabetes, relative risk multiplied by 12^24^. These individuals have also a risk of cardiovascular disease and premature death.

Diabetes was more present in males. Males had more visceral and liver fat, which combined with a lack of protective effect from estrogen, can lead to greater insulin resistance^38^. Men are biologically more susceptible to developing diabetes at a lower BMI than women. Men tend to store fat in the liver and around the waist. While women have large amounts of subcutaneous fat stored in their thighs and hips. Diabetes mellitus is associated with an excess fat located in some organs such as the liver and muscles^40,41^.

The proportion of the unemployed is higher among diabetic patients. In Finland, males with high exposure to unemployment (for three years) had a high risk of developing prediabetes and diabetes^42^, 13.1% of men compared to 10.3% among women. In this study obesity was more marked in diabetic patients as confirmed in several others studies^16,18^.

The risk factors present have already been identified in previous studies in DRC^16–18^. The abdominal obesity found in those without employment could explain the presence of diabetes in unemployed individuals.

The strengths of this study are the large sample size, the high response rate and it is the first diabetes prevalence study in this area and in the DRC that used three glycaemia in the whole sample. The limit is the difficulty of not being able to determine the amount of alcohol and tobacco consumed.

This study shows the importance of taking into account the OGTT, otherwise, there is an underestimation of cases. On the other hand, by this study, we seek to draw the attention of political and health decision-makers for the urgency of implementing disease prevention strategies.

In conclusion, diabetes prevalence in DRC, and particularly in rural areas, remains undervalued. The problem is more important than that described. It would be advised to take into account the uncounted individuals revealed by OGTT.
